# Movement Disorders Associated With Cerebral Artery Stenosis: A Nationwide Study

**DOI:** 10.3389/fneur.2022.939823

**Published:** 2022-07-14

**Authors:** Kye Won Park, Nari Choi, Eungseok Oh, Chul Hyoung Lyoo, Min Seok Baek, Han-Joon Kim, Dalla Yoo, Jee-Young Lee, Ji-Hyun Choi, Jae Hyeok Lee, Seong-Beom Koh, Young Hee Sung, Jin Whan Cho, Hui-Jun Yang, Jinse Park, Hae-Won Shin, Tae-Beom Ahn, Ho-Sung Ryu, Sooyeoun You, Seong-Min Choi, Bum Joon Kim, Seung Hyun Lee, Sun Ju Chung

**Affiliations:** ^1^Department of Neurology, Uijeongbu Eulji Medical Center, Eulji University School of Medicine, Uijeongbu, South Korea; ^2^Department of Neurology, Heavenly Hospital, Goyang, South Korea; ^3^Department of Neurology, Chungnam National University College of Medicine, Chungnam National University Hospital, Daejeon, South Korea; ^4^Department of Neurology, Gangnam Severance Hospital, Yonsei University College of Medicine, Seoul, South Korea; ^5^Department of Neurology, Wonju Severance Christian Hospital, Yonsei University Wonju College of Medicine, Wonju, South Korea; ^6^Department of Neurology, Movement Disorder Center, College of Medicine, Seoul National University Hospital, Seoul, South Korea; ^7^Department of Neurology, Kyung Hee University College of Medicine, Seoul, South Korea; ^8^Department of Neurology, Seoul National University-Seoul Metropolitan Government Boramae Medical Center and Seoul National University Medical College, Seoul, South Korea; ^9^Department of Neurology, Pusan National University Yangsan Hospital, Pusan National University School of Medicine, Yangsan, South Korea; ^10^Department of Neurology, Korea University College of Medicine, Guro Hospital, Seoul, South Korea; ^11^Department of Neurology, Gachon University Gil Medical Center, Incheon, South Korea; ^12^Department of Neurology, Samsung Medical Center, Sungkyunkwan University School of Medicine, Seoul, South Korea; ^13^Department of Neurology, Ulsan University Hospital, University of Ulsan College of Medicine, Ulsan, South Korea; ^14^Department of Neurology, Haeundae Paik Hospital, Inje University, Busan, South Korea; ^15^Department of Neurology, Chung-Ang University College of Medicine, Seoul, South Korea; ^16^Department of Neurology, Kyungpook National University Hospital, Daegu, South Korea; ^17^Department of Neurology, Dongsan Medical Center, Keimyung University, Daegu, South Korea; ^18^Department of Neurology, Chonnam National University Hospital, Gwangju, South Korea; ^19^Department of Neurology, Asan Medical Center, University of Ulsan College of Medicine, Seoul, South Korea

**Keywords:** movement disorders, intracranial artery stenosis, extracranial artery stenosis, moyamoya disease, cerebral artery stenosis

## Abstract

**Background:**

Studies of secondary movement disorder (MD) caused by cerebrovascular diseases have primarily focused on post-stroke MD. However, MD can also result from cerebral artery stenosis (CAS) without clinical manifestations of stroke. In this study, we aimed to investigate the clinical characteristics of MD associated with CAS.

**Materials and Methods:**

A nationwide multicenter retrospective analysis was performed based on the data from patients with CAS-associated MDs from 16 MD specialized clinics in South Korea, available between January 1999 and September 2019. CAS was defined as the >50% luminal stenosis of the major cerebral arteries. The association between MD and CAS was determined by MD specialists using pre-defined clinical criteria. The collected clinical information included baseline demographics, features of MD, characteristics of CAS, treatment, and MD outcomes. Statistical analyses were performed to identify factors associated with the MD outcomes.

**Results:**

The data from a total of 81 patients with CAS-associated MD were analyzed. The mean age of MD onset was 60.5 ± 19.7 years. Chorea was the most common MD (57%), followed by tremor/limb-shaking, myoclonus, and dystonia. Atherosclerosis was the most common etiology of CAS (78%), with the remaining cases attributed to moyamoya disease (MMD). Relative to patients with atherosclerosis, those with MMD developed MD at a younger age (*p* < 0.001) and had a more chronic mode of onset (*p* = 0.001) and less acute ischemic lesion (*p* = 0.021). Eight patients who underwent surgical treatment for CAS showed positive outcomes. Patients with acute MD onset had a better outcome than those with subacute-to-chronic MD onset (*p* = 0.008).

**Conclusions:**

This study highlights the spectrum of CAS-associated with MD across the country. A progressive, age-dependent functional neuronal modulation in the basal ganglia due to CAS may underlie this condition.

## Introduction

Movement disorder (MD) is considered primary when it occurs as an isolated syndrome and secondary as a symptom of various neurological disorders or systemic diseases (1). The causes of secondary MD include metabolic, infectious, traumatic, toxic, and cerebrovascular diseases. Among them, MD caused by cerebrovascular diseases is one of the most common forms, accounting for up to 22% of all secondary MDs (2).

Previous research on secondary MD caused by cerebrovascular diseases has mainly focused on post-stroke MDs (3). Post-stroke MD refers to movement complications associated with ischemic or hemorrhagic stroke leading to parenchymal destruction (4). Approximately 1–3% of patients with acute stroke develop MDs localizable to the stroke lesion (5, 6). Nevertheless, the clinical profile of post-stroke MD is diverse, both in terms of its forms and prognosis (4).

However, cerebrovascular diseases are not limited to stroke. Cerebral artery stenosis (CAS) and subsequent cerebral hypoperfusion can also cause MD even without overt parenchymal damage due to stroke. MD is also the predominant symptom of moyamoya disease (MMD), a non-atherosclerotic cause of intracranial artery stenosis. However, the clinical profile of MDs associated with arterial stenoses has not been comprehensively characterized due to the heterogeneity of MDs and arterial stenoses, as well as the difficulty in defining the association between the two conditions. These clinical entities are particularly important in the context of East Asia, where the prevalence of intracranial atherosclerosis and MMD is much higher than in Western countries (7, 8).

In this nationwide study, we aimed to characterize the clinical features of MD associated with CAS in Korean patients.

## Materials and Methods

We performed a nationwide multicenter retrospective analysis on CAS-associated MD cases collected from 16 major tertiary care hospital clinics in South Korea specializing in MDs. The 16 centers were Asan Medical Center, Chungnam National University Hospital, Seoul National University Hospital, Gangnam Severance Hospital, Seoul National University-Seoul Metropolitan Government Boramae Medical Center, Pusan National University Yangsan Hospital, Korea University College of Medicine Guro Hospital, Gachon University Gil Medical Center, Samsung Medical Center, Ulsan University Hospital, Haeundae Paik Hospital, Chung-Ang University College of Medicine, Kyung-Hee University College of Medicine, Kyungpook National University Hospital, Dongsan Medical Center, and Chonnam National University Hospital. The study was approved by the Institutional Review Board of each participating center. Informed consent of patients was waived due to the retrospective nature of this study.

Movement disorder (MD) specialists at each center reviewed the medical records of patients with CAS-associated MDs from their patient registries from January 1999 to September 2019. The inclusion criteria were as follows: (1) age of >18 years; (2) hyperkinetic or hypokinetic MD diagnosis; and (3) CAS diagnosis associated with MD, defined as >50% luminal stenosis of the anterior cerebral artery (ACA), middle cerebral artery (MCA), posterior cerebral artery (PCA), and distal or proximal internal carotid artery (ICA). The association between MD and CAS was determined by specialists according to the following criteria: (1) diagnosis of CAS preceding or coinciding with the onset of MD; (2) absence of other structural or primary neurological disorders better explaining the motor symptoms; (3) with lateralized symptoms, the localization of movement matching with the localization of CAS. The clinical information and the association between MD and CAS were cross-examined by an M.D. specialist from another center. The exclusion criteria were as follows: (1) diagnosis of primary MD during follow-up, including Parkinson's disease; (2) diagnosis of other neurological disorders that may manifest MD as a primary feature, including Wilson's disease, neurodegeneration with iron accumulation in the brain, infectious disease, etc.; (3) history of taking dopamine-blocking agents for more than a month, including neuroleptics and antiemetics.

The clinical information collected included baseline demographics, MD characteristics, CAS characteristics, and the outcome of MD. The baseline demographic data included age, sex, and risk factors for concomitant atherosclerosis, such as hypertension, diabetes mellitus, hyperlipidemia, coronary heart disease, and smoking. MD characteristics included subtype, distribution, and onset. As for the subtype, MDs were classified into one of the following: chorea (with or without ballism), dystonia, parkinsonism myoclonus, tremor or limb-shaking, and a mixed phenotype of the above. The phenotype was determined by an MD specialist using the generally accepted definitions of each phenotype. With regard to distribution, MDs were classified as focal (affecting one part of the body), unilateral (affecting the ipsilateral arm and leg), or generalized. Based on how MD symptoms developed after the patient first noticed them, the onset of MD was defined as acute (<1 week), subacute (1–4 weeks), or chronic (>4 weeks). According to the outcome of MDs, patients were divided into groups with a good outcome (self-limited, improved with medical treatment, improved with endovascular/surgical intervention) and with a poor outcome (static or progressive). The etiology of CAS was divided into atherosclerosis and MMD.

For statistical analysis, continuous variables were compared with the Student's *t*-test or Kruskal–Wallis test, as appropriate. Categorical variables were compared with the chi-square test or Fisher's exact test, as appropriate. The *p*-value of < 10.05 was considered statistically significant. All statistical analyses were performed using R v.4.1.0.

## Results

### Baseline Characteristics

A total of 96 cases were initially examined. Fifteen patients were further excluded (age of <18 in 6 patients, insufficient association between MD and CAS in 9 patients), and 81 patients were included in the final analysis.

**Figure 1:**
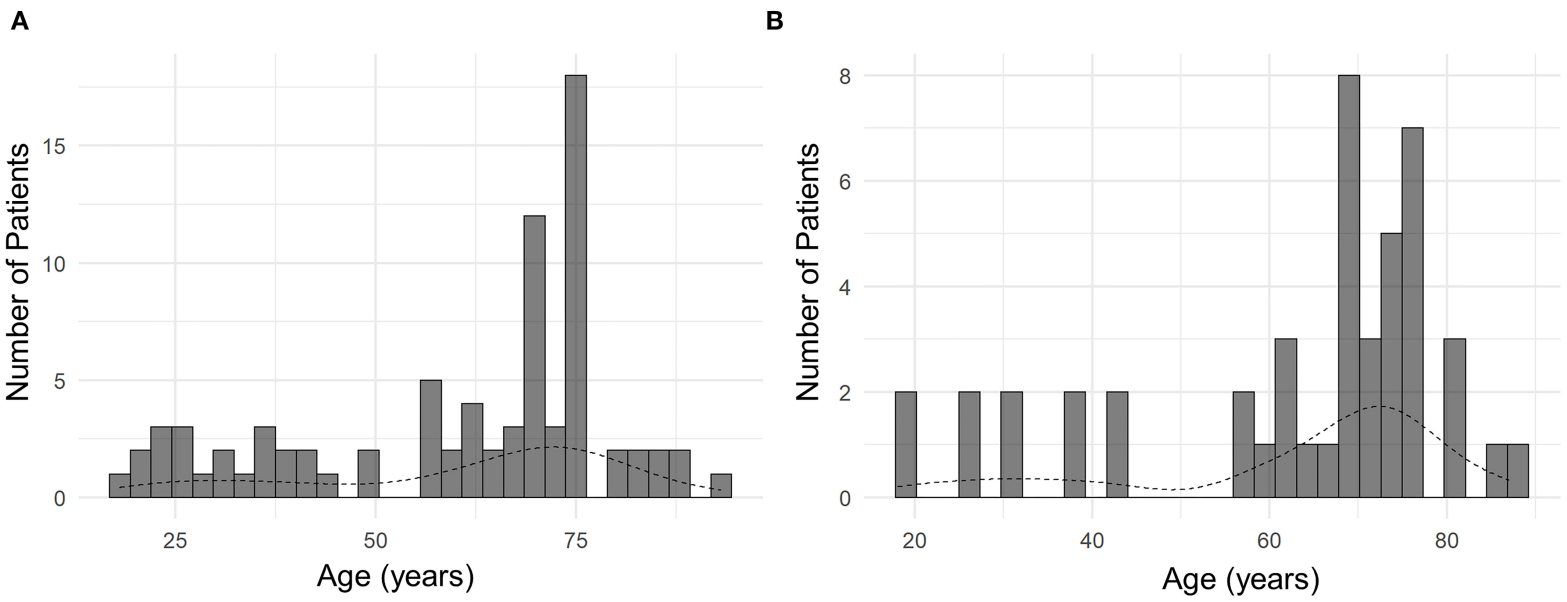
Distribution of patients by age. **(A)** All patients. **(B)** Patients with chorea.

The mean age of all patients was 60.5 ± 19.7 years, ranging from 18 to 90 years (Table 1). Bimodal age distribution was observed in all patients and the subset of patients with chorea (Figure 1). Such age distribution pattern was not observed in patients with other MDs, possibly due to a small number of patients with those MDs. The sex ratio was near 1:1, with male patients comprising 57% of the sample. Hypertension was the most common risk factor for atherosclerosis (51%), followed by diabetes (27%) and smoking (22%) (Table 1).

**Table 1:** Baseline characteristics of patients (*n* = 81).

**Characteristics**	
**Demographics**	
Age, years	60.5 ± 19.7
Sex, male	46 (57)
Risk factors for atherosclerosis	
Hypertension	41 (51)
Diabetes	22 (27)
Hyperlipidemia	13 (16)
Smoking	18 (22)
Coronary heart disease	7 (9)
**MD**	
**MD subtype**	
Chorea	46 (57)
Dystonia	7 (9)
Parkinsonism	3 (4)
Myoclonus	8 (10)
Tremor/limb-shaking	12 (15)
Mixed	5 (6)
**Distribution of MD**	
Focal	28 (35)
Unilateral	49 (60)
Generalized	4 (5)
**MD onset**	
Acute	40 (49)
Subacute	9 (11)
Chronic	32 (40)
**CAS**	
**Etiology and localization**	
Atherosclerosis	63 (78)
MCA	22 (27)
ACA	0 (0)
PCA	6 (7)
Distal ICA	6 (7)
Proximal ICA	9 (11)
Multiple stenoses	20 (25)
Moyamoya disease	18 (22)
**Acute stroke lesion**	
Present	26 (32)
Absent	55 (68)
**Associated symptoms**	
Motor weakness	25 (31)
Sensory loss	17 (21)
Dysarthria	19 (24)
Limb ataxia	4 (5)
Others	14 (17)

*Data are presented as the mean ± standard deviation or the number of patients (%). ACA, anterior cerebral artery; CAS, cerebral artery stenosis; ICA, internal carotid artery; MCA, middle cerebral artery; MD, movement disorder; PCA, posterior cerebral artery*.

### Characteristics of MDs

**Figure 2:**
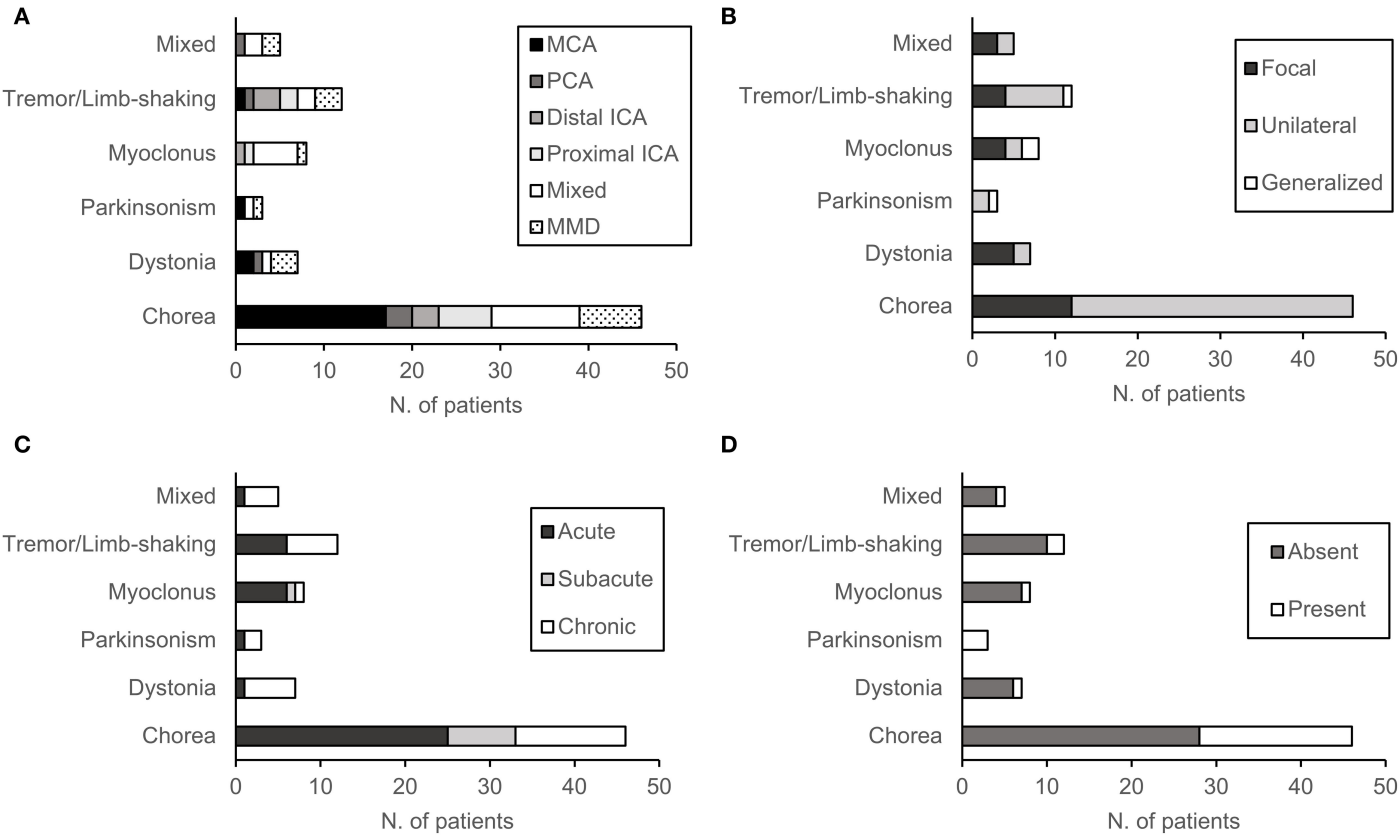
Characteristics of patients according to movement disorder phenotype: **(A)** Location and etiology of cerebral arterial stenosis (CAS), **(B)** mode of onset, **(C)** distribution of symptoms, and **(D)** the presence of acute stroke lesion.

Chorea was the most common MD (*n* = 46, 57%) followed by tremor/limb-shaking (*n* = 12, 15%), myoclonus (*n* = 8, 10%), dystonia (*n* = 7, 9%), and mixed MDs (*n* = 5). About half (*n* = 40, 49%) of the patients had acute onset of the MDs, while a substantial portion of patients (*n* = 32, 40%) had a chronic onset. Most patients had symptoms in one limb (*n* = 28, 35%) or hemibody (*n* = 49, 60%), whereas 4 patients had generalized symptoms (Table 1). As shown in Figure 2A, there was no predominance of a specific movement phenotype over the stenosis of a particular vessel. The details of each MD phenotype are described below.

In patients with chorea (*n* = 46), symptoms were either in one limb (*n* = 12, 26%) or in the hemibody (*n* = 34, 74%) (Figure 2). The onset of symptoms was acute in 25 patients (54%), subacute in 8 patients (17%), and chronic in 13 patients (29%). Atherosclerosis was the etiology of CAS in 39 patients (85%), whereas 7 patients had MMD (15%). Atherosclerosis included intracranial stenosis of the MCA (*n* = 17), PCA (*n* = 3), distal ICA (*n* = 3), proximal ICA (*n* = 6), and multiple arteries (*n* = 6). Eighteen patients had acute infarction lesions in various areas of the basal ganglia and brainstem. These lesions included a single lesion in the putamen (*n* = 4), globus pallidus (*n* = 1), thalamus (*n* = 3), and pons (*n* = 2), or multiple lesions (*n* = 9). Between choreic (n = 46) and non-choreic patients (*n* = 35), there was no statistically significant difference in the baseline demographics and MD or CAS characteristics (Supplementary Table 1).

In patients with tremor/limb-shaking (*n* = 12), the second most common form of MD, symptoms were generalized in 1 patient (Figure 2). The onset of symptoms was acute in half of the patients (*n* = 6) and chronic in the other half (*n* = 6). Atherosclerosis was the etiology of CAS in 9 tremor/limb-shaking patients, which included stenosis of the MCA (*n* = 1), PCA (*n* = 1), distal ICA (*n* = 3), proximal ICA (*n* = 2), and multiple arteries (*n* = 2). Three patients had MMD. Two patients had acute infarction lesions in the thalamus. Between patients with tremor/limb-shaking (*n* = 12) and the remaining patients with MD (*n* = 69), there was no statistically significant difference in the baseline demographics and MD or CAS characteristics (Supplementary Table 2).

In patients with myoclonus (*n* =8), the third most common form of MD, symptoms were generalized in 25% of patients (*n* = 2). Symptoms were focal in half of the patients (**n** = 4) and unilateral in two patients (Figure 2). The onset of symptoms was acute in 6 patients, subacute in one patient, and chronic in 1 patient. In myoclonic patients, the etiology of CAS was atherosclerosis of the distal ICA (*n* = 1), proximal ICA (*n* = 1), multiple arteries (*n*= 5), as well as MMD (*n* = 1). One patient had acute infarction lesions in the putamen and globus pallidus (Figure 2D).

In 7 patients with dystonia, symptoms were either focal (*n* = 5) or unilateral (*n* = 2) (Figure 2). The onset of symptoms was mostly chronic (*n* = 6), except for 1 case with an acute onset. The etiology of CAS was atherosclerosis of the MCA (*n* = 2), PCA (*n* = 1), multiple arteries (*n* = 1), as well as MMD (*n* = 3). One patient had an acute lesion in the putamen.

In three patients with parkinsonism, symptoms were either unilateral (*n* = 2) or generalized (*n* = 1) (Figure 2), and 1 patient had developed acute parkinsonism. The etiology of CAS was atherosclerosis of the MCA (*n* = 1), multiple arteries (*n* = 1), as well as MMD (*n* = 1). All three patients had acute ischemic lesions in the putamen.

Finally, there were five patients with mixed MD. Two patients had chorea and dystonia, two had chorea and tremor/limb-shaking, and one had dystonia and myoclonus. These symptoms were either focal (*n* =3) or unilateral (*n* = 2) (Figure 2). Four patients had a chronic onset of symptoms, and one patient had an acute onset. One patient had stenosis of the PCA, one had multiple intra-/extracranial atherosclerotic lesions, and two patients had MMD. One patient with chorea and dystonia had an acute ischemic lesion in the putamen.

**Figure 3:**
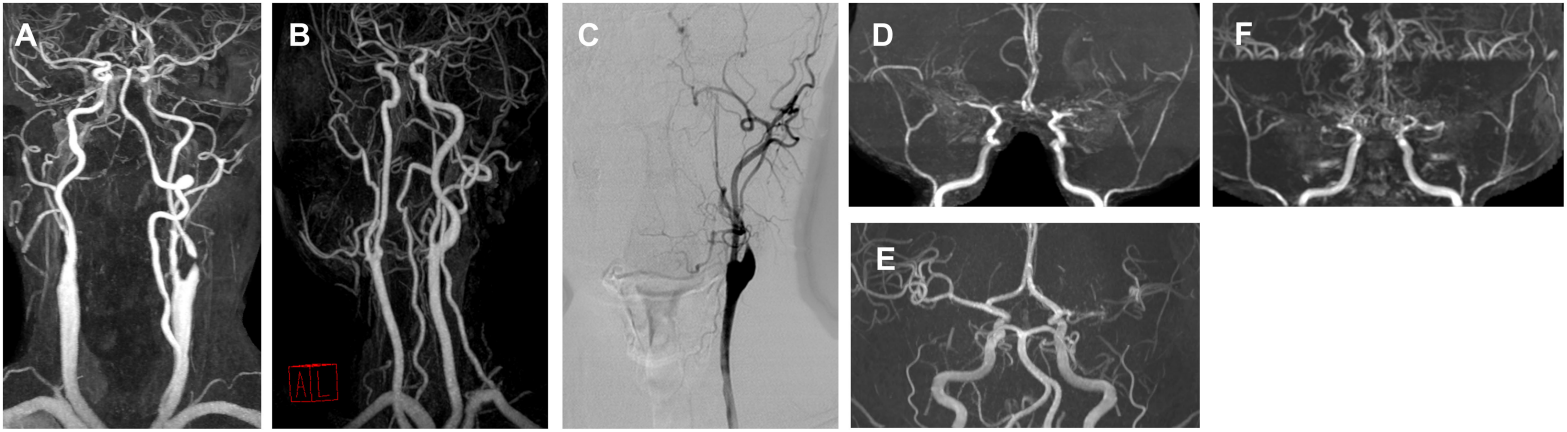
Angiography of representative cases for each movement disorder phenotype. **(A)** A 76-year-old male with right hemichorea and contralateral proximal internal carotid artery (ICA) stenosis. **(B)** A 57-year-old male with limb-shaking of the left arm and contralateral proximal ICA stenosis. **(C)** A 56-year-old male with a myoclonus of the right arm and leg and contralateral proximal ICA stenosis. **(D)** A 24-year-old male with left arm dystonia and moyamoya disease. **(E)** A 46-year-old female with right hemiparkinsonism and contralateral middle cerebral artery (MCA) stenosis. **(F)** A 28-year-old female with a mixed phenotype (left hemichorea and hemidystonia) and moyamoya disease.

The vascular status of representative cases for each movement phenotype is presented in Figure 3, and their clinical characteristics are shown in Supplementary Table 3.

### Characteristics of CAS

Overall, 78% of patients (*n* = 64) had atherosclerotic disease and the rest had MMD. Among those with atherosclerosis, 34 patients had intracranial atherosclerosis of the MCA (27%), PCA (7%), and distal ICA (7%). Nine patients (11%) had extracranial proximal ICA stenosis, and 21 patients (26%) had multiple atherosclerotic stenoses of various arteries.

Patients with MMD were significantly younger than those with atherosclerosis at symptom onset (*p* < 0.001) (Table 2). Most patients with MMD had a subacute or chronic onset of MD symptoms (*n* = 15.88%), whereas 41% (*n* = 26) of patients with atherosclerosis had an acute onset (*p* = 0.001). Only 1 patient with MMD had an overt stroke lesion at MD onset, while 25 patients with atherosclerosis (40%) also had it (*p* = 0.001). As expected, patients with atherosclerosis had significantly more risk factors for atherosclerosis than MMD patients (*p* = 0.013). However, there was no difference in the ratio of chorea between patients with atherosclerosis and MMD patients.

**Table 2:** Comparison between patients with atherosclerosis (*n* = 64) and moyamoya disease (*n* = 17).

	**Atherosclerosis (*n* = 64)**	**Moyamoya disease (*n* = 17)**	***p*-value**
**Age**	66.9 ± 14.6	36.4 ± 17.9	<0.001
**Sex**			
Male	40 (37)	6 (35)	0.082
Female	24 (63)	11 (65)	
**Number of risk factors for atherosclerosis**	2 [1-2]	0 [0–0.5]	0.013
**MD phenotype**			
Chorea	25 (40)	10 (59)	0.235
Non-chorea	39 (61)	7 (41)	
**Localization of MD**			
Focal/unilateral	60 (94)	17 (100)	0.669
Generalized	4 (6)	0 (0)	
**MD onset**			
Acute	38 (59)	2 (12)	0.001
Subacute/chronic	26 (41)	15 (88)	
**Acute stroke lesion**			
Present	25 (39)	16 (94)	0.021
Absent	39 (61)	1 (6)	

*Data are presented as the mean ± standard deviation, median [interquartile range], or the number of patients (%). MD, movement disorder*.

Acute stroke lesion was present in 26 patients (32%). Motor weakness was the most common neurological symptom associated with MD (*n* = 25, 31%), followed by dysarthria (*n* = 19, 24%) and sensory loss (*n* = 17, 21%).

### Treatment and Outcome

In 20 patients (25%), MD spontaneously improved without medical or surgical intervention. Eight patients (10%) underwent surgical treatment for CAS, and all had full resolution of their MD. Two patients with proximal ICA stenosis underwent carotid endarterectomy, and 6 patients with MMD underwent bypass surgery. The duration of post-intervention follow-up ranged from 4 to 239 months.

Fifty-three (65%) patients received medical treatment, including antichoreic, antidystonic, or antiparkinsonian medications along with antithrombotic agents. Among them, 36 patients (44%) showed improvement after pharmacological treatment, while the remaining patients (*n* = 17.21%) had symptoms that persisted.

In summary, 64 patients (79%) showed a good outcome of MD regardless of whether they received treatment or not, which was characterized by the improvement in movement symptoms. Seventeen patients (21%), however, showed no improvement in MD despite receiving medical treatment. There was no significant association between the outcome and the baseline demographic factors, including age, sex, number of risk factors for atherosclerosis, MD phenotype (chorea *vs*. non-chorea), localization of MD, etiology of CAS, location of CAS, or the presence of acute stroke lesion (Table 3). However, patients with a good outcome had a more acute onset of MD (*n* = 37, 58%) than patients with a poor outcome (*n* = 3, 18%) (*p* = 0.008).

**Table 3:** Comparison of demographics, MD characteristics, and CAS characteristics between patients with a good outcome and a poor outcome.

	**Good outcome (*n* = 64)**	**Poor outcome (*n* = 17)**	***p*-value**
**Age**	62.1 ± 18.7	54.6 ± 22.7	0.166
**Sex**			1.000
Male	36 (56)	10 (59)	
Female	28 (44)	7 (41)	
**Number of risk factors for atherosclerosis**	2 [1-2]	2 [1-2]	0.830
**MD phenotype**			1.000
Chorea	36 (56)	10 (59)	
Non-chorea	28 (44)	7 (41)	
**Localization of MD**			0.669
Focal/unilateral	60 (94)	17 (100)	
Generalized	4 (6)	0 (0)	
**MD onset**			0.008
Acute	37 (58)	3 (17)	
Subacute/chronic	27 (42)	14 (82)	
**Acute stroke lesion**			0.542
Present	45 (70)	59 (10)	
Absent	19 (30)	7 (41)	
**Etiology**			0.964
Atherosclerosis	50 (78)	14 (82)	
MMD	14 (22)	3 (18)	
**Localization of CAS**			0.114
Intracranial	37 (58)	14 (82)	
Extracranial/mixed	27 (42)	3 (17)	

*Data are presented as the mean ± standard deviation, median [interquartile range], or the number of patients (%). CAS, cerebral artery stenosis; MD, movement disorder; MMD, moyamoya disease*.

## Discussion

Given the diverse nature of CAS and hyperkinetic or hypokinetic MDs, few attempts have been made to examine the nature of MD-related CAS in a large series. Our study explored the characteristics of secondary MD associated with CAS across the country. Chorea was the most common type of MD associated with CAS. All movement phenotypes resulted from stenoses of various vascular localization without a specific predominance of the phenotype over a specific area of stenosis. However, we observed that MDs resulting from MMD appear at a younger age, and have a more chronic onset and less acute ischemic lesion compared with MDs caused by atherosclerosis. In our study, patients who received surgical treatment for arterial stenosis showed a good prognosis of MD. In patients with a good outcome of CAS-associated MD, the onset was more acute than in patients with a poor outcome.

In this study, chorea was the most frequent MD associated with CAS, present in 57% of patients. It is also the most frequent movement phenotype among post-stroke MDs, with a prevalence of 36–38% reported in previous studies (5). Traditionally, the subthalamic nucleus was considered to be a typical anatomical correlate for hemichorea and hemiballism (9). However, it has been found that various stroke lesions involved in the striato-pallido-thalamo-cortical feedback loop, including the caudate nucleus, putamen, thalamus, and subcortical white matter, also cause chorea or ballism (10–13). Moreover, there is accumulated evidence of CAS-associated chorea without overt stroke lesions (14–17). These studies uniformly demonstrated striatal hypoperfusion on neuroimaging and chorea reversal after carotid stenting or endarterectomy.

Tremor/limb-shaking was the second most common form of MD in our study. In post-stroke MDs, tremor is usually associated with lesions of the thalamus or structures of the dentato-rubro-thalamic tract or the cerebello-thalamo-cortical network (4, 18). Limb-shaking, often referred to as “limb-shaking transient ischemic attack (TIA),” was first reported in association with carotid stenosis by Miller-Fisher (19). TIA refers to transient attacks of repetitive brief limb-shaking of the leg or arm associated with carotid stenosis, which lasts from a few seconds to several days (20). Like other vascular paroxysmal dyskinesia, these clinical entities are explained by the hypoperfusion theory. A regional decrease of cerebral blood flow in the dorsofrontal and upper rolandic regions was observed during episodes of limb-shaking (21). Cases show remission of limb-shaking attacks after successful revascularization of carotid stenosis (20–23).

Myoclonus was the third most common phenotype of CAS-associated MD, followed by dystonia. Tremor/limb-shaking and myoclonus are difficult to differentiate, and limb-shaking is sometimes classified as myoclonus (22–24). Theoretically, myoclonus can be the result of any lesion involving the cortical and subcortical white matter (25). Since the basal ganglia are more vulnerable to hypoxia than these areas (26), it seems that myoclonus was not a frequent phenotype.

Dystonia in post-stroke MD is usually reported with stroke lesions in the striato-pallido-thalamo-cortical loop, including the lenticular nucleus, putamen, and thalamus (18). Dystonia is also frequently reported as a movement symptom of MMD without an overt stroke lesion, especially as a form of transient dystonia during hyperventilation (27–29). In our study, MMD was also the cause of dystonia in about half of the cases (3 out of 7).

Taken together, various hyperkinetic MDs including chorea, tremor/limb-shaking, dystonia, and myoclonus, may arise from CAS. Studies have shown hypoperfusion of the basal ganglia and the reversal of movement symptoms with successful revascularization of the stenosed vessel. In all eight patients in our study who underwent surgical treatment to restore blood supply, the remission of MD was observed. The basal ganglia, especially the striatum, are particularly vulnerable to ischemia or hypoxia (26). Hypoperfusion of the basal ganglia by CAS appears to alter the functional balance of motor circuits, leading to various hyperkinetic MDs (30). However, there was no predominance of a specific movement phenotype over a specific localization of stenosis. It can be hypothesized that the motor loop predominantly affected by hypoperfusion (e.g., dentato-rubro-thalamic loop, striato-pallido-cortical loop) determines the dominant motor phenotype.

Then how does hypoperfusion of the basal ganglia alter the motor loop? Experimental studies have shown that energy deprivation causes a surge of dopamine in the striatum (31). In other words, hypoperfusion of the basal ganglia may lead to a relative dominance of the direct pathway over the indirect pathway of the basal ganglia motor circuit. Moreover, glutamatergic activity appears to be altered in animal models of hypoxic-ischemic brain damage (32). In other words, ischemia-trigged glutamatergic excitotoxicity may contribute to the development of CAS-associated MD. In contrast to the usual clinical course of ischemic stroke, 40% of our patients had a gradual onset of symptoms, which further developed within 4 weeks. Overall, these findings suggest a progressive, ongoing functional modulation of neurons in the basal ganglia due to CAS. Such functional modulation may include dopaminergic hypersensitivity, excitotoxicity, and possibly other neurobiological processes such as neuroinflammation. These mechanisms are similar to levodopa-induced dyskinesia in Parkinson's disease (33). The fact that patients with a good outcome have a more acute onset rather than a chronic one may also be in line with the functional modulation hypothesis, suggesting that early initiation of treatment to improve blood supply prevents the perpetuation of MD before ongoing functional neuromodulation prevails. However, the reasons why hypoperfusion of the basal ganglia due to CAS causes MD in only a subgroup of patients, why this phenotype is manifested in patients with various MDs, and why chorea is the most common form of MD should be further clarified.

MD associated with MMD had several notable features. First, MMD was present in more than 20% of patients. The previous case reports on MD associated with MMD mainly present individuals from the East Asian population, which confirms the importance of our study for the region (27, 34–36). Second, the bimodal age distribution was characteristic. MD often occurs as a symptom of MMD and is a poor prognostic factor, especially in children and young adults (34, 37, 38). Moreover, a bimodal age distribution pattern is a distinct epidemiological characteristic of the MMD population (39). According to this pattern, patients with MMD were younger than patients with atherosclerosis and all patients in our study. Third, MD associated with MMD had a more chronic onset and less acute lesion compared to MD associated with atherosclerosis. In other words, synaptic plasticity changes in accordance with the changes in hypoxia that occur with age (40). This finding may support our hypothesis that functional neuronal modulation in the basal ganglia caused by CAS is progressive and age-dependent.

To the best of our knowledge, this is the first nationwide study that systematically examines heterogeneous clinical characteristics of MD associated with CAS. Our study has several limitations. First, the clinical data were collected by an MD specialist who reviewed the patient registry at their respective center. Due to the inherent limitation of a retrospective study, the source of patient registries varied across the centers, including those from MD-oriented registries and those from stroke-oriented registries. Patients who had only minimal MD treated by non-MD specialists could have been overlooked, which might affect epidemiological estimates. Second, the evidence for an association between MD and CAS was based solely on clinical judgment. Since there is no definitive diagnostic work to ascertain the causality of CAS with MD, we have developed clinical criteria ourselves to determine this association. Third, although patients who underwent surgical treatment for CAS showed a good prognosis of MD, this study would not be sufficient to provide recommendations on the treatment guidelines for CAS-associated MD due to its retrospective design. Further prospective studies are warranted.

In conclusion, this study analyzed the nature of CAS associated with MD on a nationwide scale. Progressive, ongoing functional neuronal modulation in the basal ganglia due to CAS may lead to MD. Further studies should focus on the epidemiology of these clinical entities and the identification of the pathogenic mechanism underlying the heterogeneity of CAS-associated MD.

## Data Availability Statement

The raw data supporting the conclusions of this article will be made available by the authors, without undue reservation.

## Ethics Statement

The studies involving human participants were reviewed and approved by Asan Medical Center, Chungnam National University Hospital, Seoul National University Hospital, Gangnam Severance Hospital, Seoul National University-Seoul Metropolitan Government Boramae Medical Center, Pusan National University Yangsan Hospital, Korea University College of Medicine Guro Hospital, Gachon University Gil Medical Center, Samsung Medical Center, Ulsan University Hospital, Haeundae Paik Hospital, Chung-Ang University College of Medicine, Kyung-Hee University College of Medicine, Kyungpook National University Hospital, Dongsan Medical Center, and Chonnam National University Hospital. Written informed consent for participation was not required for this study in accordance with the national legislation and the institutional requirements.

## Author Contributions

KP and NC contributed to the conceptualization, data curation, formal analysis, investigation, visualization, and writing of the original draft. MB, DY, J-HC, and SL contributed to the data curation, investigation, and visualization. EO, CL, H-JK, J-YL, JL, S-BK, YS, JC, H-JY, JP, H-WS, T-BA, H-SR, SY, and S-MC contributed to the data curation, methodology, supervision, and review and editing of the original draft. BK contributed to the review and editing of the original draft. SC contributed to the conceptualization, data curation, formal analysis, funding acquisition, investigation, methodology, supervision, and review and editing of the original draft. All authors contributed to the article and approved the submitted version.

## Funding

This study was supported by a grant from the Korea Healthcare Technology R&D Project, Ministry of Health and Welfare, Republic of Korea (HI19C0256).

## Conflict of Interest

The authors declare that the research was conducted in the absence of any commercial or financial relationships that could be construed as a potential conflict of interest.

## Publisher's Note

All claims expressed in this article are solely those of the authors and do not necessarily represent those of their affiliated organizations, or those of the publisher, the editors and the reviewers. Any product that may be evaluated in this article, or claim that may be made by its manufacturer, is not guaranteed or endorsed by the publisher.

## References

[1] Youssef PE Mack KJ Flemming KD. Mayo Clinic Neurology Board ReviewClinical Neurology for Initial Certification and MOC. Classification and Approach to Movement Disorders: Oxford University Press (2015). 10.1093/med/9780190244927.003.0024

[2] Netravathi M Pal P Indira Devi B. A clinical profile of 103 patients with secondary movement disorders: correlation of etiology with phenomenology. Eur J Neurol. (2012) 19:226–33. 10.1111/j.1468-1331.2011.03469.x 21777351

[3] Siniscalchi A Gallelli L Labate A Malferrari G Palleria C Sarro GD. Post-stroke movement disorders: clinical manifestations and pharmacological management. Curr Neuropharmacol. (2012) 10:254–62. 10.2174/157015912803217341 23449883 PMC3468879

[4] Kwon DY. Movement disorders following cerebrovascular lesions: etiology, treatment options and prognosis. J Mov Disord. (2016) 9:63–70. 10.14802/jmd.16008 27240807 PMC4886206

[5] Ghika-Schmid F Ghika J Regli F Bogousslavsky J. Hyperkinetic movement disorders during and after acute stroke: the lausanne stroke registry. J Neurol Sci. (1997) 146:109–16. 10.1016/S0022-510X(96)00290-0 9077506

[6] Alarcón F Zijlmans JC Dueñas G Cevallos N. Post-stroke movement disorders: report of 56 patients. J Neurol Neurosurg Psychiatry. (2004) 75:1568–74. 10.1136/jnnp.2003.011874 15489389 PMC1738792

[7] Chen HX Wang LJ Yang Y Yue FX Chen LM Xing YQ. The prevalence of intracranial stenosis in patients at low and moderate risk of stroke. Ther Adv Neurol Disord. (2019) 12:1756286419869532. 10.1177/1756286419869532 31447935 PMC6693021

[8] Kim JS Bonovich D. Research on intracranial atherosclerosis from the East and west: why are the results different? J Stroke. (2014) 16:105–13. 10.5853/jos.2014.16.3.105 25328869 PMC4200588

[9] Lee MS Marsden CD. Movement disorders following lesions of the thalamus or subthalamic region. Mov Disord. (1994) 9:493–507. 10.1002/mds.870090502 7990845

[10] Chang JH Seo W-K Park M-H Lee J-M Kwon D-Y Koh S-B. Generalized chorea induced by an unilateral anterior cerebral artery territorial infarction. J Mov Disord. (2009) 2:37–9. 10.14802/jmd.09009 24868351 PMC4027690

[11] Barinagarrementeria F Vega F Del Brutto OH. Acute hemichorea due to infarction in the corona radiata. J Neurol. (1989) 236:371–2. 10.1007/BF00314386 2795109

[12] Guida D Biraschi F Francione G Orzi F Fantozzi LM. Hemichorea–hemiballism syndrome following a thrombo-embolic striatal infarction. Neurol Sci. (2013) 34:599–601. 10.1007/s10072-012-1098-6 22532104

[13] Pantano P Cesare SD Ricci M Gualdi GF Sabatini U Piero VD. Hemichorea after a striatal ischemic lesion: evidence of thalamic disinhibition using single-photon emission computed tomography: a case report. Mov Disord. (1996) 11:444–7. 10.1002/mds.870110417 8813228

[14] Kim DW Ko Y Jang SH Yoon SJ Oh G-S Lee SJ. Acute hemichorea as an unusual presentation of internal carotid artery stenosis. J Mov Disord. (2013) 6:17–20. 10.14802/jmd.13004 24868420 PMC4027650

[15] Parees I Pujadas F Hernandez-Vara J Lorenzo-Bosquet C Cuberas G Munuera J. Reversible hemichorea associated with extracranial carotid artery stenosis. J Neurol Sci. (2011) 300:185–6. 10.1016/j.jns.2010.08.068 20888600

[16] Noda K Ishimoto R Hattori N Okuma Y Yamamoto T. Hemichorea improvement following endarterectomy for internal carotid artery stenosis. J Neurol Sci. (2016) 371:45–7. 10.1016/j.jns.2016.10.019 27871446

[17] Morigaki R Uno M Suzue A Nagahiro S. Hemichorea due to hemodynamic ischemia associated with extracranial carotid artery stenosis: report of two cases. J Neurosurg. (2006) 105:142–7. 10.3171/jns.2006.105.1.142 16871890

[18] Mehanna R Jankovic J. Movement disorders in cerebrovascular disease. Lancet Neurol. (2013) 12:597–608. 10.1016/S1474-4422(13)70057-7 23602779

[19] Fisher CM. Concerning recurrent transient cerebral ischemic attacks. Can Med Assoc J. (1962) 86:1091. 13893225 PMC1849196

[20] Rosenbaum S Ovesen C Futrell N Krieger DW. Inducible limb-shaking transitory ischemic attacks: a video-documented case report and review of the literature. BMC Neurol. (2016) 16:78. 10.1186/s12883-016-0601-8 27215317 PMC4878005

[21] Tatemichi TK Young WL Prohovnik I Gitelman DR Correll JW Mohr JP. Perfusion insufficiency in limb-shaking transient ischemic attacks. Stroke. (1990) 21:341–7. 10.1161/01.STR.21.2.341 2406995

[22] Muraga K Suda S Nagayama H Okubo S Abe A Aoki J. Limb-shaking TIA: cortical myoclonus associated with ICA stenosis. Neurology. (2016) 86:307–9. 10.1212/WNL.0000000000002293 26683641

[23] Yoon Y Kim JS. Limb-shaking TIA: an asterixis. Neurology. (2013) 81:931–2. 10.1212/WNL.0b013e3182a351bd 23902703

[24] Kim H Byun JS Hallett M Shin H-W. Multifocal myoclonus as a manifestation of acute cerebral infarction recovered by carotid arterial stenting. J Mov Disord. (2017) 10:64–6. 10.14802/jmd.16040 28122425 PMC5288662

[25] Zutt R van Egmond ME Elting JW van Laar PJ Brouwer OF Sival DA. A novel diagnostic approach to patients with myoclonus. Nat Rev Neurol. (2015) 11:687–97. 10.1038/nrneurol.2015.198 26553594

[26] Fugate JE. Anoxic-ischemic brain injury. Neurol Clin. (2017) 35:601–11. 10.1016/j.ncl.2017.06.001 28962803

[27] Kumar S Sharma S Jhobta A Sood RG. Dystonia an unusual presentation in pediatric moyamoya disease: imaging findings of a case. J Pediatr Neurosci. (2016) 11:115–7. 10.4103/1817-1745.187629 27606018 PMC4991150

[28] Lyoo CH Oh SH Joo J-Y Chung T-S Lee MS. Hemidystonia and hemichoreoathetosis as an initial manifestation of moyamoya disease. Arch Neurol. (2000) 57:1510–2. 10.1001/archneur.57.10.1510 11030805

[29] Bakdash T Cohen AR Hempel JM Hoagland J Newman AJ. Moyamoya, dystonia during hyperventilation, and antiphospholipid antibodies. Pediatr Neurol. (2002) 26:157–60. 10.1016/S0887-8994(01)00367-8 11897484

[30] Utter AA Basso MA. The basal ganglia: an overview of circuits and function. Neurosci Biobehav Rev. (2008) 32:333–42. 10.1016/j.neubiorev.2006.11.003 17202023

[31] Büyükuysal RL Mete B. Anoxia-induced dopamine release from rat striatal slices: involvement of reverse transport mechanism. J Neurochem. (1999) 72:1507–15. 10.1046/j.1471-4159.1999.721507.x 10098855

[32] Dang Y Wang X. Evaluation of altered glutamatergic activity in a piglet model of hypoxic-ischemic brain damage using ^1^H-MRS. Dis Markers. (2020) 2020:8850816. 10.1155/2020/8850816 33029259 PMC7532412

[33] Bezard E Brotchie JM Gross CE. Pathophysiology of levodopa-induced dyskinesia: Potential for new therapies. Nat Rev Neurosci. (2001) 2:577–88. 10.1038/35086062 11484001

[34] Baik JS Lee MS. Movement disorders associated with moyamoya disease: a report of 4 new cases and a review of literatures. Mov Disord. (2010) 25:1482–6. 10.1002/mds.23130 20629162

[35] Xu J Li S Rajah GB Zhao W Ren C Ding Y. Asymmetric lenticulostriate arteries in patients with moyamoya disease presenting with movement disorder: three new cases. Neurol Res. (2020) 42:665–9. 10.1080/01616412.2020.1782121 32586217

[36] Lee JY Kim SK Wang KC Chae JH Cheon JE Choi JW. Involuntary movement in pediatric moyamoya disease patients: consideration of pathogenetic mechanism using neuroimaging studies. Childs Nerv Syst. (2014) 30:885–90. 10.1007/s00381-013-2339-6 24337519

[37] Kuroda S Houkin K. Moyamoya disease: current concepts and future perspectives. Lancet Neurol. (2008) 7:1056–66. 10.1016/S1474-4422(08)70240-0 18940695

[38] Kim S-K Cho B-K Phi JH Lee JY Chae JH Kim KJ. Pediatric moyamoya disease: an analysis of 410 consecutive cases. Ann Neurol. (2010) 68:92–101. 10.1002/ana.21981 20582955

[39] Kim JS. Moyamoya disease: epidemiology, clinical features, and diagnosis. J Stroke. (2016) 18:2–11. 10.5853/jos.2015.01627 26846755 PMC4747069

[40] Oberman L Pascual-Leone A. Changes in plasticity across the lifespan: cause of disease and target for intervention. Prog Brain Res. (2013) 207:91–120. 10.1016/B978-0-444-63327-9.00016-3 24309252 PMC4392917

