# Supplementary material for: Movement Disorders Associated With Cerebral Artery Stenosis: A Nationwide Study
Source: Front Neurol. 2022 Jul 14;13:939823. doi: 10.3389/fneur.2022.939823 (PMC9330487; doi:10.3389/fneur.2022.939823)
Supplement: Supplementary file 1 [file Data_Sheet_1.PDF]

Table S1. Comparison between choreic (n = 46) and non-choreic (n = 35) patients

	Chorea (n = 46)	Non-chorea (n = 35)	P value
**Age**	62.7 ± 18.3	57.7 ± 21.4	0.264
**Sex**			0.525
**Male**	25 (52)	22 (63)	
**Female**	22 (48)	13 (37)	
**Number of atherosclerotic risk factors**	1 [0–2]	2 [0–2]	0.627
**Location of MD**			0.067
**Focal/unilateral**	46 (100)	31 (89)	
**Generalized**	0 (0)	4 (11)	
**Onset of MD**			0.424
**Acute**	25 (54)	15 (43)	
**Subacute/chronic**	21 (46)	20 (57)	
**Acute stroke lesion**			0.189
**Present**	18 (39)	8 (23)	
**Absent**	28 (61)	27 (77))	
**Aetiology**			0.235
**Atherosclerosis**	39 (85)	25 (29)	
**Moyamoya disease**	7 (15)	10 (71)	
**Location of CAS**			0.803
**Intracranial**	30 (65)	21 (60)	
**Extracranial/mixed**	16 (35)	14 (40)	

Table S2. Comparison between tremor/limb-shaking (*n* = 12) and other (*n* = 69) patients

	Tremor/limb-shaking (*n* = 12)	Others (*n* = 69)	*P* value
**Age**	62.6 ± 17.2	60.1 ± 20.2	0.695
**Sex**			0.287
**Male**	9 (75)	37 (54)	
**Female**	3 (25)	32 (46))	
**Number of atherosclerotic risk factors**	1 [0–2]	1 [0–2]	0.984
**Location of MD**			1.000
**Focal/unilateral**	11 (92)	66 (96)	
**Generalized**	1 (8)	3 (4)	
**Onset of MD**			1.000
**Acute**	6 (50)	34 (49)	
**Subacute/chronic**	6 (50)	35 (51)	
**Acute stroke lesion**			0.365
**Present**	2 (17)	24 (35)	
**Absent**	10 (83)	45 (65)	
**Aetiology**			1.000
**Atherosclerosis**	9 (75)	55 (80)	
**Moyamoya disease**	3 (25)	14 (20)	
**Location of CAS**			1.000
**Intracranial**	8 (67)	43 (62)	
**Extracranial/mixed**	4 (33)	26 (38)	

Table S3. Clinical summary of the representative cases of each movement phenotype presented in Figure 3

	Case A	Case B	Case C	Case D	Case E	Case F
**MD phenotype**	Chorea	Tremor/limb-shaking	Myoclonus	Dystonia	Parkinsonism	Mixed (chorea, dystonia)
**Age**	76	57	56	24	46	28
**Sex**	M	M	M	M	F	F
**Atherosclerotic risk factors**	None	Hypertension	Smoking	Hypertension, diabetes, smoking	None	None
**Location of MD**	Unilateral, right	Focal, left arm	Unilateral, right	Focal, left arm	Unilateral, right	Unilateral, left
**Onset of MD**	Acute	Acute	Subacute	Chronic	Chronic	Chronic
**Etiology of CAS**	Atherosclerosis	Atherosclerosis	Atherosclerosis	Moyamoya disease	Atherosclerosis	Moyamoya disease
**Location of CAS**	Proximal ICA, left	Proximal ICA, right	Proximal ICA, left	Bilateral MCA	Left MCA	Bilateral MCA
**Acute Stroke lesion**	Absent	Present, MCA-PCA border zone	Absent	Absent	Absent	Absent
**Associated symptoms**	None	Hemiparesis, left	None	None	None	None
**Prognosis**	Spontaneous resolution	Spontaneous resolution	Improved with surgery	Improved with surgery	Improved with medical treatment	Persisted
**Treatment**	-	-	Extra-intracranial arterial bypass surgery	Extra-intracranial arterial bypass surgery	Levodopa, aspirin	Tetrabenazine
